# “Better at home”: Mixed methods report of intricacies in pediatric febrile neutropenia management

**DOI:** 10.1002/cam4.7106

**Published:** 2024-03-20

**Authors:** Eleanor T. Smeallie, Sung W. Choi, Rajen Mody, Timothy C. Guetterman, Charles N. Nessle

**Affiliations:** ^1^ Department of Pediatrics University of Michigan Ann Arbor Michigan USA; ^2^ Division of Pediatric Hematology Oncology University of Michigan Ann Arbor Michigan USA; ^3^ Rogel Comprehensive Cancer Center University of Michigan Ann Arbor Michigan USA; ^4^ Department of Family Medicine, Mixed Methods Program University of Michigan Ann Arbor Michigan USA; ^5^ Fogarty International Center National Institute of Health Bethesda Maryland USA

**Keywords:** cancer, care delivery, children, hospital discharge, infectious disease, qualitative

## Abstract

**Introduction:**

Many febrile neutropenia (FN) episodes are low risk (LR) for severe outcomes and can safely receive less aggressive management and early hospital discharge. Validated risk tools are recommended by the Children's Oncology Group to identify LR FN episodes. However, the complex dynamics of early hospital discharge and burdens faced by caregivers associated with the FN episode have been inadequately described.

**Methods:**

An adapted quality‐of‐life (QoL) survey instrument was administered by a convergent mixed methods design; qualitative and quantitative data from two sources, the medical record and the mixed methods survey instrument, were independently analyzed prior to linkage and integration. Code book was informed by conceptual framework; open coding was used. Mixed methods analysis used joint display of results to determine meta‐inferences.

**Results:**

Twenty‐eight patient–caregiver dyads participated with a response rate of 87%. Of the 27 FN episodes, 51.8% (14/27) were LR and 40.7% (11/27) had an early hospital discharge. The LR and early hospital discharge groups had higher mean QoL scores comparatively. Meta‐inferences are reciprocal influencers and expand the complex situation; FN negatively affects the entire family, and the benefits of hospital management were outweighed by risks and worsened symptoms, so an individualized approach to management and care at home was preferred.

**Conclusion:**

Early discharge of LR FN episodes positively impacts QoL, yet risk‐stratified management for FN is intricately complex. Optimal FN management should prioritize the patient's overall health; shared decision‐making is recommended and can improve care delivery. These results should be confirmed in a larger, more heterogeneous population.

## 1 INTRODUCTION

Febrile neutropenia (FN) is a complication of chemotherapy in children with cancer.^1^, ^2^ However, not all FN episodes present the same risk for severe outcomes and carefully identified patients can safely receive less aggressive management.^3^, ^4^ Children with low‐risk (LR) FN can safely receive oral antibiotics at home, most successful when performed in a step‐down manner with close outpatient follow‐up.^5^, ^6^, ^7^, ^8^ Febrile neutropenia guidelines from Children's Oncology Group (COG) suggested six different risk stratification tools for oncologists.^9^, ^10^ Until recently, research focused on the safety and efficacy of various models and inadequate attention was given to the patient and caregivers' experience.^11^

Children's hospitals have recently implemented risk‐stratified approaches to FN management.^12^, ^13^ To deliver FN management at home, caregivers and providers agreed a family‐oriented support infrastructure is ideal.^14^, ^15^ Caregivers are interested in FN management at home, recognize the value of provider continuity after discharge,^16^ and envision improvements in their quality‐of‐life (QoL) as a result.^15^ Caregivers favor shared decision‐making in FN management and early hospital discharge, but face difficulty during discussions with providers due to the tension between risk stratification value and anecdotal risk perception.^15^, ^17^ Few qualitative evaluations of FN episodes describe the experiences and burdens of dyads and further in‐depth descriptions, especially in a North American cohort, are needed.^15^

Our institution implemented a risk‐stratified management approach for the early hospital discharge of eligible LR FN episodes per international and COG guidelines.^9^, ^18^ In response to the call for caregivers' inclusion in active FN projects and studies,^14^ this report expands the description of the experiences patient/caregiver dyads diagnosed with FN by the novel utilization of a mixed methods survey instrument.

## 2 METHODS

### 2.1 Study design and planning

We previously evaluated a COG recommended risk‐stratification tool^9^, ^18^ modified with additional high‐risk (HR) criteria and procalcitonin.^19^ This observational, convergent mixed methods study used a joint display of integrated mixed methods data collection during study planning, informed by a theoretical framework.^20^ The joint display table illustrated how the mixed quantitative and qualitative data from medical chart abstraction and the mixed methods survey instrument were conceptually and thematically related. The study received ethical approval from the University of Michigan institutional review board. This report follows the standards for reporting qualitative research guidelines.^21^

### 2.2 Setting and definitions

Fever neutropenia was defined as an absolute neutrophil count (ANC) less than 0.5 K/μL, or 1 K/μL with anticipation that it would decrease; and a fever of ≥38°C sustained over 1 h or a single temperature >38.3°C.^22^ Our tertiary, referral center follows COG guidelines in the treatment of fever neutropenia by administration of empiric antipseudomonal parental antibiotics.^9^ A COG recommended risk tool^18^, ^23^ modified with more risk criterion and serial procalcitonin was used.^19^ Described in a prior publication, children were eligible for early hospital discharge after one night of hospital observation if they met the LR criteria; the decision for early discharge was shared between the patient/caregiver and medical team.^19^ Episodes were HR if they did not meet LR criteria and continued to receive standard admission.^19^ (eTable 1).

### 2.3 Mixed methods survey instrument development and participants

The original survey instrument was modified^6^ so the 13 quantitative items shared a common Likert scale of “far below average” to “far above average,” and 8 qualitative items were added to thematically expand upon the quantitative items (eTable 2). General oncology dyads under age 21 were eligible after hospital discharge for FN where the first fever was observed in the outpatient setting approached via phone or in person during the study period. Over a 5‐month period (January–May 2022) and early in the implementation of FN risk stratification, 31 patient/caregiver (1:1) dyads were approached 7 days after hospital discharge with a response rate of 87%; dyads were instructed to collaboratively respond on their most recent FN episode. Recruitment continued to thematic saturation (*n* = 28), the point at which continued data collection stopped adding complexity for codes or themes.^24^ Verbal consent was obtained by standardized script. Surveys were emailed or administered by research assistants via a virtual platform (Qualtrics, Provo, UT).

### 2.4 Mixed methods survey instrument analysis

The theoretical framework and joint display of integrated data collection^20^ informed identification of reoccurring themes by an open coding method.^25^ Discrepancies were discussed, then the final coded transcript was uploaded into NVivo Pro 11 (QSR International, Melbourne, Australia). A thematic analysis^24^, ^25^ of the qualitative data was conducted. Categorical variables were summarized by means, while coefficient alpha^26^ described the internal consistency reliability of quantitative items using Excel software v.2209.

### 2.5 Medical chart analysis

REDCap secure database^27^ stored the medical chart abstraction data. Descriptive statistical analysis was performed using Excel software v.2209. The sample size was determined by the estimated thematic saturation point for the mixed methods survey instrument. Therefore, a bivariate power analysis was not performed.

### 2.6 Mixed methods analysis

After independent analyses of each data type, a joint display analysis^28^ integrated both types of data to determine agreement and discrepancy, guided and informed by the integrated joint display of data collection.^20^ Joint displays of results illustrated the data, demonstrated linkage between data type and origin, and then interpretation produced meta‐inferences and significance for each theme. A joint display figure was generated (Draw.io software, v20.7.4) to depict the complex relationships of results in the mixed methods survey instrument.

## 3 RESULTS

### 3.1 Quantitative results

Quantitative results were analyzed from two separate data sources: medical chart abstraction and mixed methods survey items.

#### 3.1.1 Medical chart abstraction

Analysis for the 27 FN episodes identified 51.8% (14/27) as LR episodes and 48.1% (13/27) as HR episodes with an early hospital discharge in 40.7% (11/27) of FN episodes (eTable 3 and eTable 4). Of the 11 early hospital discharges, 81.8% (9/11) were LR and 18.2% (2/11) were HR; there were 0 intensive care or blood stream infections in this group. The FN episodes in the standard admission group included 31.3% (5/16) LR episodes and 68.8% (11/16) HR episodes, with a median length‐of‐stay of 3 days (range 1–8 days). The standard admission group had one blood stream infection and one intensive care admission observed in separate HR FN episodes.

#### 3.1.2 Quantitative mixed method survey items

Results from the quantitative items of the mixed methods survey instrument are depicted in eTable 5. Coefficient alpha^26^ was 0.782. The mean score for LR FN group (2.8) was close to the HR FN group (2.7), but the mean score for the early hospital discharge group (3) was higher than the standard admission group (2.6). All groups had mean scores below or far below average in all items referencing their child. The LR FN group kept up better with household tasks compared to the HR FN group (2.7 vs. 2.1) and spent time more time with their partner (2.8 vs. 2.1). Compared to the standard admission group, the early hospital discharge group was more satisfied with their care (4.6 vs. 4.1), kept up with household tasks better (3 vs. 1.9) and described their child as more independent (3.1 vs. 2.3).

### 3.2 Qualitative results

Thematic analysis yielded qualitative results (*n* = 28) from responses to the 8 open‐ended items in the mixed methods survey instrument.^21^, ^22^ Responses were generally a few sentences in length. Three main themes were identified: the family unit experiences burden, the preference for home FN management, and the desire for a comprehensive approach to FN management (Table 1).

**TABLE 1:** Thematic analysis of qualitative question items from mixed methods survey instrument.

Theme	Subthemes
Family units experience burden	Families require significant support during FN episodes Febrile neutropenia episodes are emotionally difficult for caregivers
Home management is preferred	Hospitalization comes with risks Care at home is preferred
Comprehensive approach to management	Addressing all symptoms is important Optimal management for fever neutropenia

#### 3.2.1 Family units experience burden

Participants described that the global strain of caring for a child with cancer was worsened by FN and disrupted their entire family unit. Caregivers (CG) spent less time with their other children, partners, and needed extra support (e.g., extended family and friends) due to FN‐related hospitalization:

[There was] an extra week apart due to another hospital stay in between chemo treatments. [The] kids really started to miss each other. (CG 25; HR FN, Standard Admission)

Two subthemes were caregivers' need for extra help and a worsened emotional state during FN episodes. Caregivers relied on their support system to balance the responsibilities of caring for their child in the hospital and family at home:

[The FN hospital admission] kept our family apart for 3 extra days. My aunt had to watch my boys as my husband had to work. (CG 04; LR FN, Standard Admission)

Due to an early hospital discharge for a LR FN episode, one caregiver highlighted family members were not negatively impacted: *Because of this new rule, we did not have to stay in the hospital very long which was great!* (CG 05; LR FN, Early Hospital Discharge)

Participants disclosed emotional challenges, commonly described as elevated stress and emotional strain:

Honestly having your child have cancer affects you […] This episode in particular is like a race within a greater race. It was exhausting. (CG 01; LR FN, Standard Admission)

#### 3.2.2 Home management is preferred

Many participants considered the impact that treatment location, at home or hospital admission, had on various aspects of their daily life and FN management. Participants valued collaboration with the pediatric oncology team to ensure safe, quality care in both locations. However, they recognized the negative and positive aspects of both treatment locations. Positive remarks about treatment location referenced care at home, while negative comments were associated with hospital admission:

When my child is admitted to the hospital, a lot of her activities that she likes to do get cut off. At home she is sunshine and rainbows, she has energy to spare, wants to play. She does not like being contained in a box‐like room for days on end. She has a great appetite at home, sleeps well, likes to laugh and be silly. (CG 17; HR FN, Standard Admission)

Subthemes were the risks associated with hospitalization and an overall preference for care at home. The major concerns of care in the hospital were exposure to hospital‐based infections, acquiring illnesses from other patients, and risk of medical harm:

Being able to stay home when possible is important to me because I worry about exposure at the hospital. (CG 07; HR FN, Standard Admission)

Despite concern about infection, some caregivers preferred the proximity to the hospital medical team:

We got [a]round the clock care so her blood count numbers usually are better maintained within the hospital then at home. (CG 14; HR FN, Standard Admission)

Beyond mitigated risks of hospitalization, care at home improved social, emotional, and familial factors:

She is upset and struggles with her appetite while in the hospital … She definitely does better at home (CG 08; HR FN, Standard Admission)

Although most caregivers favored care at home for the benefit of their child's social and emotional functioning, one caregiver noted, *“[Her] interaction mentally and socially [is] better because change in environment and people [at the hospital]”* (CG 15; LR FN, Early Hospital Discharge).

#### 3.2.3 Comprehensive approach to management

Medical management discussion highlighted the subjective risks and challenges of hospital admission. Many responses stressed the importance of the management of associated symptoms and comorbidities exacerbated by FN. Overall, participants indicated a desire for tailored care delivery unique to each patient and episode.

Once the blood cultures are growing and my child has received the 24‐hour antibiotic, I am more than confident that I can go home with her and monitor. […]Sitting in the ER for 5‐7 hours is obnoxious and exposes my child even further. A lot of the neutropenic treatment would be better in the oncology offices. (CG 11; HR FN, Early Hospital Discharge)

Two sub‐themes were identified: symptom management and FN specific treatment. Caregivers noted FN episodes aggravated their child's other symptoms: decreased activity, altered mood, impaired sleep, and reduced appetite:

My child is typically happy and easy going, when she has a neutropenic fever, she is very clingy and upset. (CG 06; LR FN, Standard Admission)

Caregivers desired individualized care during FN episodes, the value of antipyretics, and preference for an oncologist at presentation:

There needs to be different treatments for children who present with neutropenia fevers with no signs of infection. They were giving her fluids and antibiotics for an infection that wasn't there … (CG 09; LR FN, Standard Admission)

When medical management was tailored to the patient, they spoke favorably about their FN treatment:

My child's team changed up the nausea and pain meds and that has resulted in us being able to stay home during the most recent [neutropenic] spell. (CG 11; HR FN, Early Hospital Discharge)

### 3.3 Mixed method results

**FIGURE 1:**
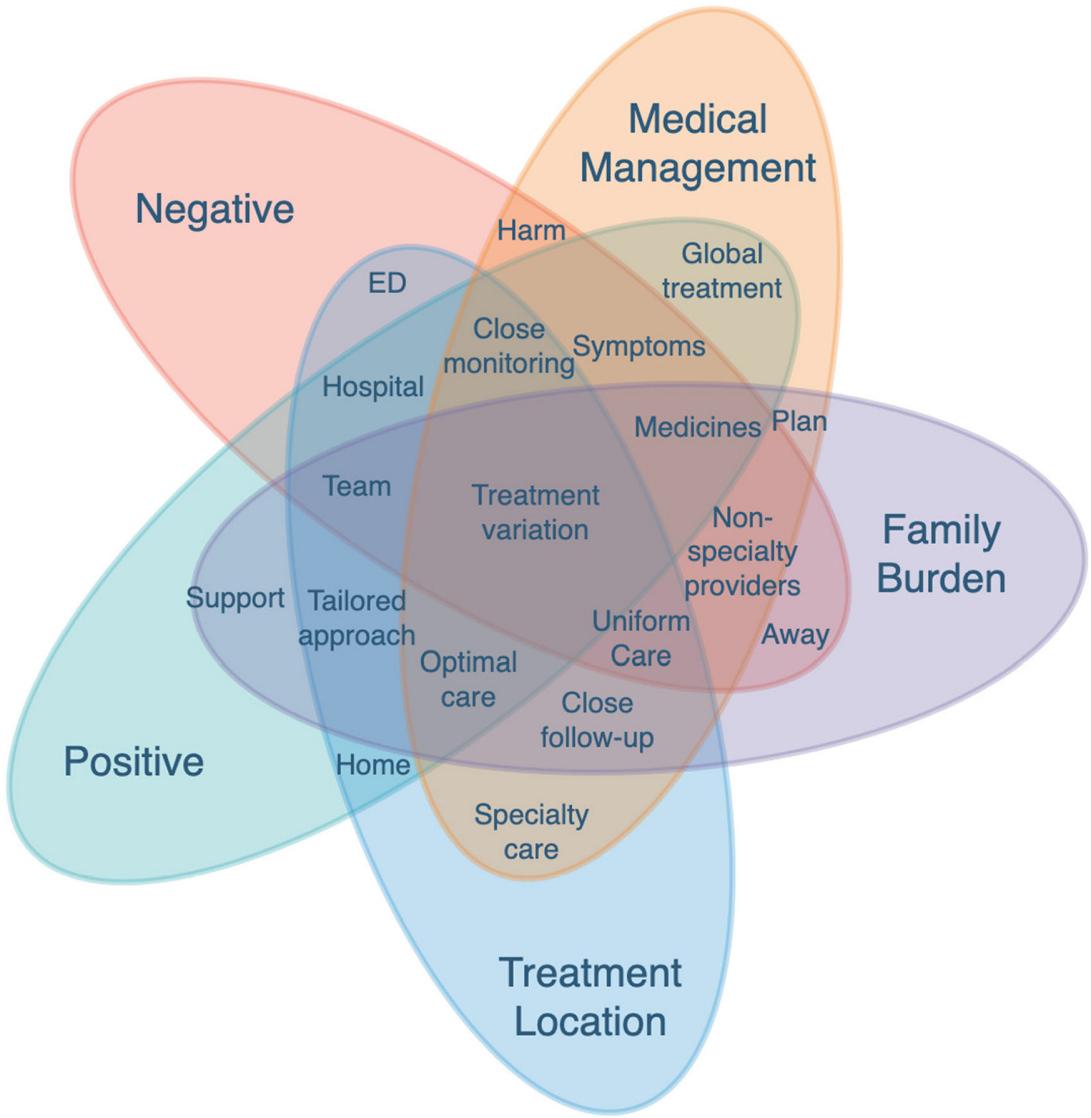
Visual joint display of factors discussed in mixed method survey instrument. Overlapping colors indicate factors with multiple sentiments (i.e., close monitoring was discussed both negatively and positively within the treatment location and medical management constructs).

Independent analysis of the quantitative and qualitative data occurred before the merger and linkage of data in the integrated joint display tables of analysis (Tables 2, 3, 4). The meta‐inferences from the integrated mixed methods analysis are presented in the joint display tables, along with relevant qualitative quotations, quantitative graphs, and meta‐inferences. The mixed methods analysis highlighted: (1) FN episodes negatively impact the child's entire family unit; (2) caregivers appreciate the benefits and risks acquired by the treatment location and ascertain that at‐home treatment at home would be most beneficial when empowered by the treatment team; (3) pediatric cancer caregivers desire a comprehensive, global approach to FN management for their child and family. A joint display figure was then constructed to improve description of the complex relationships of the facets involved in a risk‐stratified management approach for FN (Figure 1).

**TABLE 2:** Mixed methods joint display of family burden.

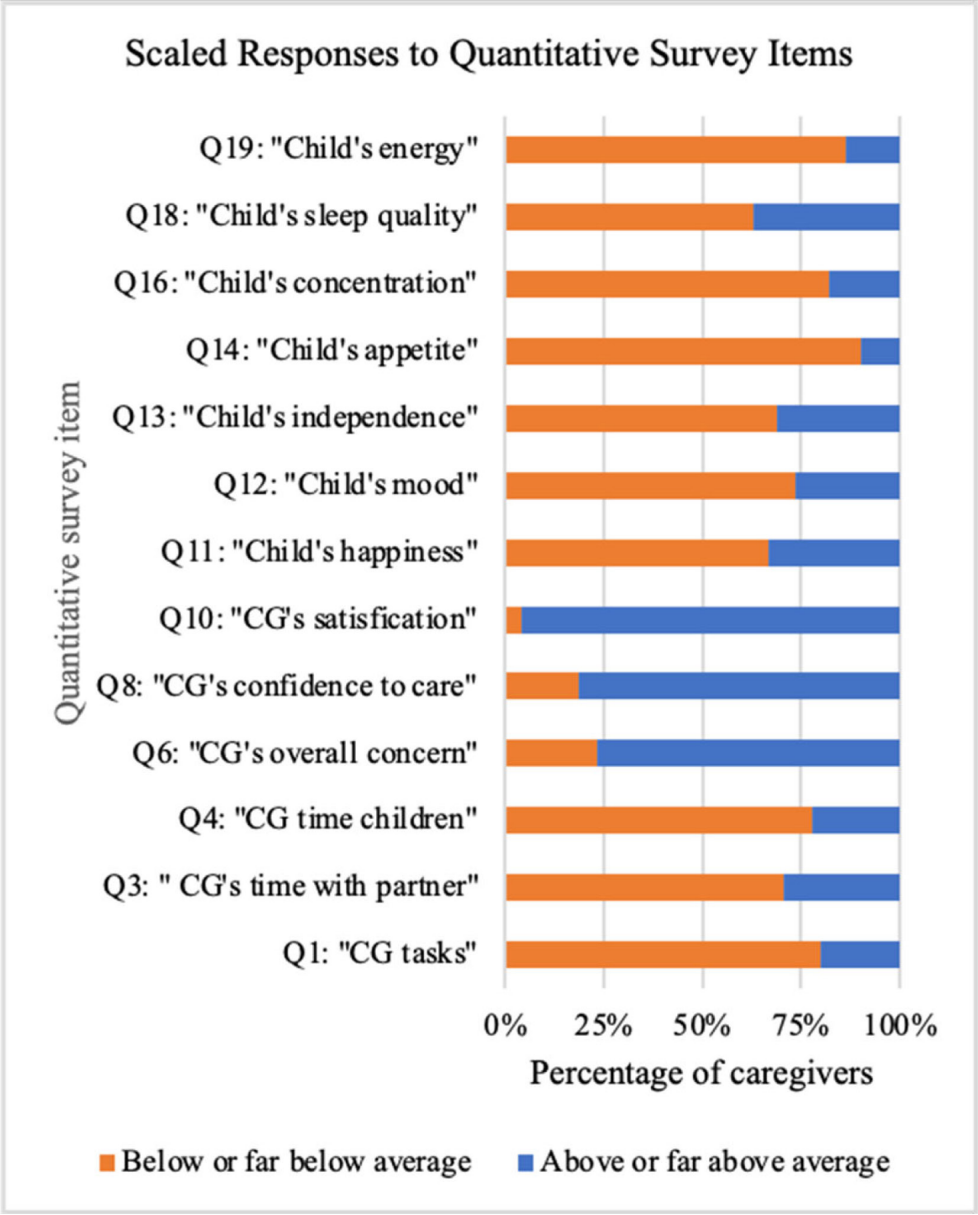

The meta‐inferences were reciprocally related: the negative familial burden influenced the treatment location which informed the medical management of FN episodes. The dynamic, complex relationships between meta‐inferences and the positive and negative factors in FN management are best illustrated in the visual joint display (Figure 1). The visual joint display described factors (e.g., “hospital” and “team”) discussed in both negative and positive ways, and factors with either a negative sentiment (e.g., “non‐specialty providers” and “harm”) or positive sentiment (e.g., “home” and “global treatment”). For example, within “treatment location,” both favorable and unfavorable aspects of hospital admission were expressed.

## 4 DISCUSSION

### 4.1 Summary of main findings

The mixed methods survey instrument results indicated that implementation of safe, effective risk‐stratification for early discharge of LR FN patients is preferred and benefits patients, caregivers, and their families. The first meta‐inference (Table 2) described the significant strain the entire family unit endured during FN episodes. Although their support system helped with logistics, caregivers described significant emotional burden. Further, the second meta‐inference (Table 3) demonstrated the preference for home management due to improved patient symptoms, decreased family burden, and reduced exposure to hospital‐based infections, which outweighed the benefits of hospital management (e.g., monitoring and access to immediate intervention). Somatic and psychological symptoms were exacerbated in the hospital, and optimal FN management should consider these comorbidities (Table 4). The risk stratified management approach and decision for early hospital discharge by shared decision making is intricately complex as facets of management seem to have dynamic relationships (Figure 1).

**TABLE 3:** Mixed methods analysis of treatment location.

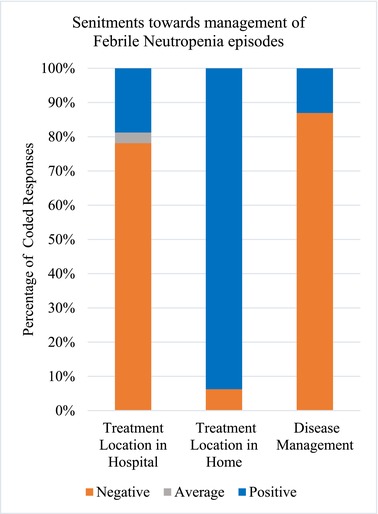

**TABLE 4:** Mixed methods analysis of medical management.

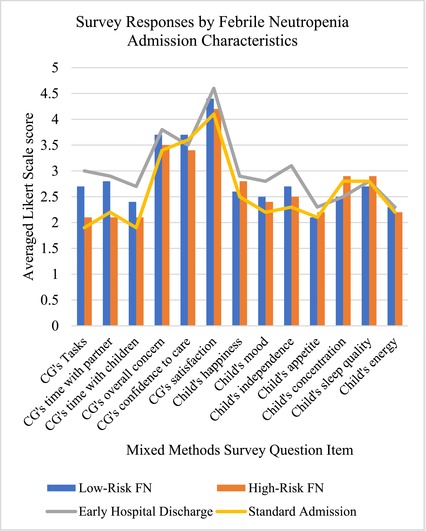

The results of this report align with current literature. Caregivers desire treatment team consideration of the family burden and stress related to FN episodes.^14^, ^15^, ^16^, ^29^ Another study evaluated the health‐related quality‐of‐life (HRQoL) of patient/caregiver dyads, and correlated decreased HRQoL with FN episode onset.^30^ Strong preference for at‐home care was attributed to increased hospitalization risks, decreased familial burden, and improved patient/caregiver QoL.^14^, ^15^, ^16^, ^29^, ^30^ Caregivers desired a comprehensive, individualized approach to FN management that considers the patient's overall health (emotional, social, and somatic).^14^, ^15^, ^16^, ^29^ Our findings further support the inclusion of families, caregivers, and overall patient well‐being in shared decision‐making of FN episodes,^14^, ^16^, ^29^ which may improve navigation of the complexities within a risk stratified management approach and individualization of FN management to best meet the child and family needs.

In contrast to our findings, one study's quantitative survey indicated that, despite the perceived improvement in HRQoL for early discharge and outpatient care, caregivers and patients preferred hospital care.^31^ However, early discharge of LR FN was not offered.^31^ Although few studies implemented risk stratification for FN, a preference for management at home was reported, as negative aspects of hospital care were improved by outpatient management.^14^, ^15^, ^16^, ^29^, ^30^ Further, several studies focused on the LR FN patient population and excluded HR FN patients.^14^, ^15^, ^16^, ^29^, ^30^, ^31^

### 4.2 Strengths and weaknesses

This study expands current literature by its mixed methods research design, which facilitated illustration of the complex relationships between the positive and negative factors involved in a risk‐stratified management approach for FN episodes. Prior studies analyzed either qualitative^14^, ^16^, ^31^ or quantitative data,^30^ with one study that analyzed both types of data separately without mixed methods analysis, which limited appreciation of the complexities.^15^ Second, incorporation of patient/caregiver dyads responded to the call to include caregivers in active FN projects and studies,^14^ which were considered in the quality improvement study. Furthermore, this study evaluated the influence early discharge of LR FN had on dyad experiences after the clinical implementation of risk stratification^18^, ^19^ and showed an overall trend of QoL improvement in the LR and early discharge group. Lastly, this report addressed the need to describe the FN experience in a North American cohort^15^ as current literature is based in the United Kingdom and Australia.^14^, ^15^, ^16^, ^29^, ^30^

A limitation of this study stems from the sample size of participants, which limited the quantitative analysis of medical data from medical chart abstraction. Further, the observational study design and single‐site recruitment may restrict the study population's experiences due to possible selection bias. Survey responses were not proctored, and collaborative responses cannot be guaranteed. Respondents were mainly Caucasian and non‐Hispanic, a general characterization of our institution's patient population but fails to represent a diverse cohort. We did not collect socioeconomic status, psychosocial factors, or level of education, which may impact result interpretation. Despite these limitations, our results build upon and align with existing literature which upholds the external reliability of our findings.

### 4.3 Implications

The results of the mixed methods analysis indicate the multifaceted burden of FN management faced by families is complex and may be improved by a risk‐stratified management approach. Early hospital discharge with oral antibiotics should be considered in LR FN management.^3^, ^8^, ^9^, ^32^ The safe, effective implementation of risk‐stratified FN management may avoid prolonged hospital admissions for eligible children.

Effective communication between patient/caregiver and medical team would improve care delivery. A comprehensive approach to management was desired and the medical team should facilitate the dialogue of FN treatment strategies, which will positively impact both the patient and caregiver.^14^, ^16^ Caregivers reported positive experiences about the care received during hospital admission but also desired increased communication about length of stay, medications, and FN treatment options.

The importance of caregiver emotional well‐being in the optimization of patient health applies more broadly to cancer care.^33^ Emotional and resilience‐based interventions may enhance caregiver well‐being, which reciprocally benefits patients’ level of care and health.^33^, ^34^ Fever neutropenia episodes distress caregivers; however, these emotional difficulties are not unique to FN episodes apply to other caregivers of patients with cancer.^33^

## 5 CONCLUSION

Fever neutropenia negatively impacts everyone within the family; despite the benefits of hospital care, caregivers recognized home management improved the burdens associated with the FN episode. While a risk‐stratified management approach to FN is complex, an individualized approach with shared decision‐making between caregivers and health care professionals will improve care delivery. Although this report focused on FN episodes, the results may apply more broadly; FN episodes represent just one of many arduous aspects of the cancer journey.

Our work highlights the need for further mixed methods descriptions of cancer care to include patients and caregivers in management decisions. Improved characterization of patient and family burden would influence shared decision‐making. Future investigations may consider further evaluation of the subgroups by risk stratification (LR to HR) and disposition (early discharge to standard hospitalization) in a larger heterogeneous population.

## AUTHOR CONTRIBUTIONS

**Eleanor T. Smeallie:** Formal analysis (supporting); visualization (equal); writing – original draft (equal); writing – review and editing (equal). **Sung W. Choi:** Conceptualization (equal); formal analysis (equal); supervision (equal); validation (equal); writing – review and editing (equal). **Rajen Mody:** Conceptualization (equal); investigation (equal); supervision (lead); validation (equal); writing – review and editing (equal). **Timothy C. Guetterman:** Formal analysis (equal); methodology (lead); supervision (equal); validation (equal); visualization (equal); writing – review and editing (equal). **Charles N. Nessle:** Conceptualization (equal); data curation (lead); formal analysis (lead); investigation (lead); methodology (equal); project administration (equal); resources (lead); supervision (equal); visualization (equal); writing – original draft (lead); writing – review and editing (lead).

## FUNDING INFORMATION

Fogarty International Center and the National Cancer Institute (NCI) of the National Institutes of Health, Mentoring in patient‐oriented research, National Heart Lung and Blood Institute, and Mixed Methods Program, Department of Family Medicine, University of Michigan.

## CONFLICT OF INTEREST STATEMENT

The authors declare no competing interests.

## Supporting information


**Supplementary S1** eTable1: Setting and management of pediatric fever neutropenia.
eTable 2: Mixed methods survey instrument^a^_._
eTable 3: FN^a^ episode characteristics by hospital admission.
eTable 4: Demographics of FN^a^ episodes.
eTable 5: Mean caregiver responses of quantitative survey items from mixed methods instrument by FN risk and hospital admission type.

## Data Availability

Deidentified quantitative and qualitative data collected as part of this study are available upon reasonable request to the corresponding author.
