# Corrigendum to “Better at home”: Mixed methods report of intricacies in pediatric febrile neutropenia management

**DOI:** 10.1002/cam4.7373

**Published:** 2024-06-25

Smeallie ET, Choi SW, Mody R, Guetterman TC, Nessle CN. “Better at home”: Mixed methods report of intricacies in pediatric febrile neutropenia management. Cancer Med. 2024 Mar;13(6):e7106. doi: 10.1002/cam4.7106. PMID: 38506249; PMCID: PMC10952020.

The tables in the results section (Table 2: Mixed methods joint display of family burden; Table 3: Mixed methods joint display of treatment location; Table 4: Mixed methods joint display of medical management) were originally published only in partial format. Qualitative results and meta‐inferences from mixed methods analysis were omitted during production. The three corrected tables are shown below.

**TABLE 2:** Mixed methods joint display of family burden.

Meta‐inference: Febrile neutropenia episodes negatively impact the child's entire family unit.	
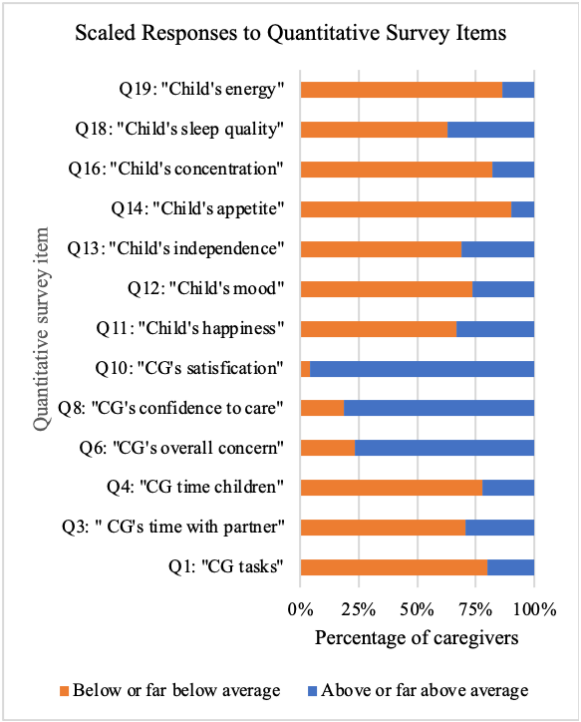	*Significance: Caregivers describe the multifaceted burden impressed upon their family during FN episodes. While they seek assistance from nuclear and extended family member to meet many logistical needs, they also describe the negative emotions associated with FN episodes.*
	**Subtheme: Families require significant support during FN episodes**
	“Each episode is very tough on our other two children. We try to keep their lives as normal as possible, but they feel neglected when we are at the hospital.”—CG 23; High‐Risk FN, Standard Admission “[I] had to enlist help from my mother and sister to take care of our youngest child during admission.”—CG 20; Low‐Risk FN, Early Hospital Discharge
	**Subtheme: Febrile neutropenia episodes are emotionally difficult for caregivers**
	“My biggest fear is that she will give up fighting because she is tired of being kept in the hospital.”—CG 17; High‐Risk FN, Standard Admission “Watching my 2‐year‐old be sick is the worst thing ever.”—CG 22; Low‐Risk FN, Early Hospital Discharge “It's definitely scary to see my child go through all of this.”—CG 02; High‐Risk FN, Standard Admission
Graph 1: The horizontal bar graph depicts the number of caregiver responses per each quantitative item from the mixed methods survey instrument. Responses per item were scaled as “below or far below average,” compared to “above or far above average”; responses recorded as “average” were omitted in this bar graph.	

**TABLE 3:** Mixed methods joint display of treatment location.

Meta‐inference: Caregivers appreciate the benefits and risks acquired by the treatment location and ascertain that treatment at home would be most beneficial when empowered by the outpatient treatment team.	
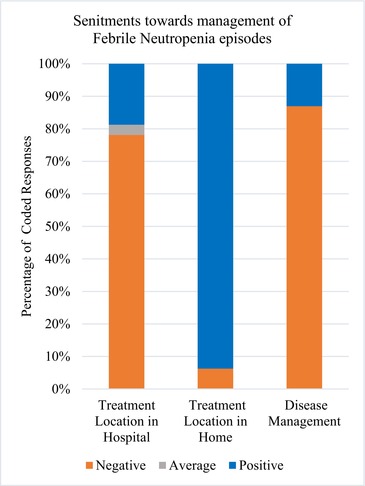	*Significance: Caregivers recognize the value of close monitoring and immediate access to interventions by hospital admission; however, this value is outweighed by the benefits from treatment at home, such as improvement in symptoms, decreased burden, and fewer exposures.*
	**Subtheme: Hospitalization come with risks**
	“At hospitals we are at higher risk for hospital based infections, etc., and we are around a whole lot of more people on a daily basis at the hospital.”—CG 14; High‐Risk FN, Standard Admission “When we go to the ER we [often] have to tell the ER docs not to [do] things due to our child being an oncology patient. This is super frustrating. They should know ‐ no rectal temps, no catheter, etc.”—CG 11; High‐Risk FN, Early Hospital Discharge
	**Subtheme: Care at home is preferred**
	**“**The fact that during a pandemic having extended time with chances of being infected [by] numerous people […] knowing that one chance meeting could put 3 out of the 4 family members at risk for severe outcomes or death. I would rather have her home if possible.”—CG 15; Low‐Risk FN, Early Hospital Discharge “In the hospital‐ just letting them rest more and not be bothered. Wishing that I could take care of him at home just like they do in the hospital with video chat visits.”—CG 13; High‐Risk FN, Standard Admission
Graph 2: The vertical bar graph depicts the percentage of codes “treatment location in hospital,” “treatment location in home,” and “disease management” with the applied negative and positive sentiments for each. Caregivers commonly provided negative statements toward disease management [87%; (20/23)] and treatment location in hospital [78%; (25/32)] while they frequently gave positive statements [94%; (15/16)] to treatment location at home.	

**TABLE 4:** Mixed methods joint display of medical management.

Meta‐inference: During FN episodes, caregivers of children with cancer desire a comprehensive, global approach to the management for their child and family	
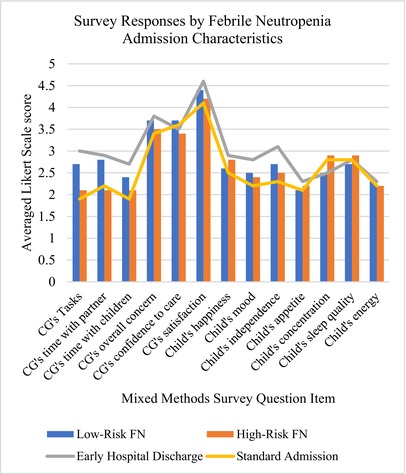	*Significance: Caregivers recognize that while timely administration of antibiotics and infection monitoring are important, they also appreciate FN exacerbates other symptoms (somatic and psychological) which are equally as important as infection monitoring.*
	**Subtheme: Addressing all symptoms is important**
	“I hate to see her suffering by pokes, taking medicine with side effects, and having pain. [My child] is now scared to tell me when something is bothering her, she fears having to go back to the hospital.”—CG 21; Low‐Risk FN, Early Hospital Discharge “Mood and appetite are always worse in the hospital.”—CG 26; High‐Risk FN, Early Hospital Discharge
	**Subtheme: Optimal management for fever neutropenia**
	“I wish there was something different that could be used to treat fevers other than [acetaminophen] when children can't have [ibuprofen] on chemo.”—CG 09; Low‐Risk FN, Standard Admission “Figuring out the right combination/timing of meds requires full focus. While admitted, the Mott staff greatly helped with this,”—CG 01; Low‐Risk FN, Standard Admission
Graph 3: The mixed vertical bar and line graph depicts the mean Likert scale responses for each quantitative item from the mixed methods survey instrument (see eTable 2) arranged by risk subgroup (low‐risk FN, high‐risk FN) and by admission type (early hospital discharge, standard admission). Compared to high‐risk FN and standard admission type, caregivers of children which were low‐risk FN or early hospital discharge type were more likely to report higher scores on the quantitative survey items.	

We apologize for this error.

