# Supplementary material for: “Better at home”: Mixed methods report of intricacies in pediatric febrile neutropenia management
Source: Cancer Med. 2024 Mar 20;13(6):e7106. doi: 10.1002/cam4.7106 (PMC10952020; doi:10.1002/cam4.7106)
Supplement: Supplementary file 1 — Supplementary S1 eTable1: Setting and management of pediatric fever neutropenia. eTable 2: Mixed methods survey instrumenta . eTable 3: FNa episode characteristics by hospital admission. eTable 4: Demographics of FNa episodes. eTable 5: Mean caregiver responses of quantitative survey items from mixed methods instrument by FN risk and hospital admission type. [file CAM4-13-e7106-s001.docx]

**Table of Contents: Supplementary Material**

Page 1: Table of Contents

Page 2: eTable 1

Page 3: eTable 2

Page 4: eTable 3

Page 5: eTable 4

Page 6: eTable 5

**eTable 1**

| eTable 1: Setting and management of pediatric fever neutropenia |
| --- |
| Setting: Our academic, pediatric referral center manages approximately 150 new cancer diagnoses and referrals annually, designated as a phase 1/2 clinical trial site by the COG conducting hematopoietic stem cell transplants (HSCT), chimeric antigen receptor therapy (CAR-T), and general oncology clinical trials. |
| Definitions: Fever neutropenia was defined as an absolute neutrophil count (ANC) less than 0.5 K/µL, or 1 K/µL with anticipation that it would decrease; and a fever of ≥38°C sustained over one hour or a single temperature >38.3°C. |
| Risk stratification: Our institution internally evaluated a clinical decision risk tool recommended by the COG, modified with more clinical risk criterion and serial procalcitonin. An episode where the first fever was detected outpatient was low-risk (LR) if the episode met clinical LR criteria at presentation in the emergency department and first morning of inpatient observation, and serial procalcitonin <0.4 ng/mL. Episodes which did not meet LR criteria or any procalcitonin ≥0.4 ng/mL were high-risk. Risk stratification and early hospital discharge was not considered in HSCT patients or episodes where the first fever was detected inpatient. |
| Risk criterion: Low-risk criterion were age older than 1 year, and not with Down Syndrome; procalcitonin <0.4 ng/mL at presentation and first morning inpatient observation; an anticipated neutropenia under 7 days defined as: initial outpatient presentation, no history of acute myelogenous leukemia or Non-Hodgkin lymphoma, no HSCT within the last year, no CAR-T within 6 months, acute leukemia must be in remission and not in induction or active relapse; there must be absence of severe comorbidity at presentation defined as: absence of hypotension requiring ≥40 ml/kg fluid boluses with 4 hours of presentation, no tachypnea or sustained hypoxia (spO2 <92%), no altered mental status, no concern for focal infection requiring intravenous antibiotics, no bacteremia, no mucositis requiring intravenous narcotics, no refractory vomiting or moderate/severe abdominal pain, no grade 2 organ dysfunction; there must be no other reason for admission, no social concern for medical adherence to follow-up, and the ability to tolerate oral antibiotics must be present |
| Inpatient management: All FN episodes where the first fever was detected outpatient were admitted to the hospital for initial management. High-risk episodes were not recommended for early discharge and instead continued with a standard hospital admission in which anti-pseudomonal antibiotics, typically a 4^th^ generation cephalosporin, continued through ANC recovery >0.2 K/µL or 5 days duration, whichever is less, if no microbiologically or clinically defined infection was diagnosed, per institutional clinical practice guidelines. |
| Outpatient management: Low-risk episodes were eligible for early hospital discharge after one night inpatient observation. The decision for early hospital discharge was shared between the patient/caregiver and the inpatient medical team. The patient would receive oral fluoroquinolone, typically levofloxacin, before discharge to complete a total 5-day course. After discharge, the patient/caregiver dyad would have an outpatient virtual follow-up visit with an oncology provider, then daily phone calls from outpatient oncology skilled nursing staff through day 5. |

**eTable 2**

| eTable 2: Mixed methods survey instrument^a^ |
| --- |
| Q1: I can keep up with work and/or household tasks. |
| *Q2: What are the extra resources and help required to keep your family functioning during fever neutropenia episodes?* |
| Q3: I am able to spend time with my partner (if applicable) |
| Q4: I am able to spend time with my other children and/or family. (if applicable) |
| *Q5: How did this fever neutropenia episodes affect members in your family?* |
| Q6: What is your overall level of concern for your child’s health and condition during this fever neutropenia episode? |
| *Q7: How has your child’s illness emotionally affected you?* |
| Q8: During this fever neutropenia episode, I am confident in my ability to care for my child. |
| *Q9: What is it like caring for your child when the have fever neutropenia?* |
| Q10: I am satisfied with the overall care my child received by medical personnel |
| Q11: What is your child’s overall happiness and well-being? |
| Q12: How would you describe your child’s mood? |
| Q13: How would you describe your child’s level of independence? |
| Q14: How would you describe your child’s appetite? |
| *Q15: How are symptoms different when your child is at home versus in the hospital?* |
| Q16: How would you describe your child’s interest or concentration? |
| *Q17: How have school and work been affected by your child’s illness?* |
| Q18: How would you describe your child’s quality of sleep? |
| Q19: How would you describe your child’s activity or energy level? |
| *Q20: In your opinion, what is the most important value when your child has fever neutropenia?* |
| *Q21: Is there anything else you would like to share about caring for your child during fever neutropenia?* |
| Qualitative survey items are denoted by *Italicized* description^a^ |

**eTable3**

| eTable 3: FN^a^ episode characteristics by hospital admission | | | |
| --- | --- | --- | --- |
| Variable | **Total FN^a^ Episodes** | **Early Discharge** | **Standard Admission** |
| FN^a^ Episodes | 27 | 11 (40.7%) | 16 (59.3%) |
| Low risk | 14 (51.9%) | 9 (81.8%) | 5 (31.3%) |
| High Risk | 13 (48.2%) | 2 (18.2%) | 11 (68.8%) |
| SLOS^b^ | 20 (74.1%) | 11 (100%) | 9 (56.3%) |
| Median LOS^c^ [range] | 2 days  [1-8 days] | 1 day  [1-3 days] | 3 days [1-8 days] |
| PICU^d^ | 1 (3.7%) | 0 (0%) | 1 (6.3%) |
| MDI^e^ | 8 (29.6%) | 3 (27.3%) | 5 (31.3%) |
| BSI^f^ | 1 (3.7%) | 0 (0%) | 1 (6.3%) |
| URI^g^ | 5 (18.5%) | 3 (27.3%) | 2 (12.5%) |
| Fungal | 0 (0%) | 0 (0%) | 0 (0%) |
| C. diff^h^ | 0 (0%) | 0 (0%) | 0 (0%) |
| UTI^i^ | 0 (0%) | 0 (0%) | 0 (0%) |
| Abbreviations: FN^a^ = fever neutropenia; SLOS^b^ = short length-of-stay ≤3 days; LOS^c^ = length-of-stay; PICU^d^ = pediatric intensive care unit; MDI^e^ = microbiologically defined infection; BSI^f^ = blood stream infection; URI^g^ = upper respiratory infection; C. diff^h^ = clostroides difficile; UTI^i^ = urinary tract infection | | | |

**eTable 4**

| eTable 4: Demographics of FN^a^ episodes | |
| --- | --- |
| Variable | **Number of episodes** |
| Oncologic Diagnosis |  |
| AML^b^ | 1 |
| ALL^c^/lymphoma | 6 |
| Burkitt Lymphoma | 2 |
| Non-Hodgkin Lymphoma | 1 |
| Non-CNS^d^ solid tumor | 16 |
| Other Malignancy | 1 |
| Race |  |
| White or Caucasian | 24 |
| Black or African American | 3 |
| America Indian/Alaskan Native | 0 |
| Native Hawaiian or other Pacific Islander | 1 |
| Asian | 1 |
| Other | 0 |
| Unknown | 0 |
| Ethnicity |  |
| Hispanic | 1 |
| Non-Hispanic | 26 |
| Age |  |
| <1 year | 0 |
| 1-4 years | 10 |
| 5-9 years | 4 |
| 10-14 years | 4 |
| 15-19 years | 5 |
| >19 years | 4 |
| Gender |  |
| Male | 7 |
| Female | 20 |
| Abbreviations: FN^a^= fever neutropenia; AML^b^= acute myelogenous leukemia; ALL^c^= acute lymphoblastic leukemia; CNS^d^= central nervous system | |

**eTable5**

| **eTable 5: Mean caregiver responses of quantitative survey items from mixed methods instrument by FN risk and hospital admission type** | | | | | |
| --- | --- | --- | --- | --- | --- |
| **Quantitative Survey Item** | **Low-Risk FN** | **High-Risk FN** | **Early Discharge** | **Standard Admission** | **All Groups** |
| Number of respondents | 15 | 13 | 12 | 16 | 28 |
| Q1:I can keep up with work and/or household tasks. | 2.7 | 2.1 | 3 | 1.9 | 2.4 |
| Q3:I am able to spend time with my partner (if applicable) | 2.8 | 2.1 | 2.9 | 2.2 | 2.5 |
| Q4:I am able to spend time with my other children and/or family. (if applicable) | 2.4 | 2.1 | 2.7 | 1.9 | 2.3 |
| Q6:What is your overall level of concern for your child's health and condition during this fever neutropenia episode? | 3.7 | 3.5 | 3.8 | 3.4 | 3.6 |
| Q8: During this fever neutropenia episode, I am confident in my ability to care for my child. | 3.7 | 3.4 | 3.5 | 3.6 | 3.6 |
| Q10:I am satisfied with the overall care my child received by medical personnel | 4.4 | 4.2 | 4.6 | 4.1 | 4.3 |
| Q11:What is your child's overall happiness and well being? | 2.6 | 2.8 | 2.9 | 2.5 | 2.7 |
| Q12:How would you describe your child's mood? | 2.5 | 2.4 | 2.8 | 2.2 | 2.5 |
| Q13:How would you describe your child's level of independence? | 2.7 | 2.5 | 3.1 | 2.3 | 2.6 |
| Q14:How would you describe your child's appetite? | 2.1 | 2.2 | 2.3 | 2.1 | 2.2 |
| Q16:How would you describe your child's interest or concentration? | 2.5 | 2.9 | 2.5 | 2.8 | 2.7 |
| Q18:How would you describe your child's quality of sleep? | 2.7 | 2.9 | 2.8 | 2.8 | 2.8 |
| Q19:How would you describe your child's activity or energy level? | 2.3 | 2.2 | 2.3 | 2.2 | 2.2 |
| Average mean scores for each category | 2.8 | 2.7 | 3.0 | 2.6 | 2.8 |
| Key: The continuous color scale indicates Likert scale responses; far below average (1) is indicated by dark yellow, average (3) is indicated by light yellow, far above average (5) is indicated by dark blue. | | | | | |
